# A Schistosome cAMP-Dependent Protein Kinase Catalytic Subunit Is Essential for Parasite Viability

**DOI:** 10.1371/journal.pntd.0000505

**Published:** 2009-08-25

**Authors:** Brett E. Swierczewski, Stephen J. Davies

**Affiliations:** Department of Microbiology and Immunology, Uniformed Services University of the Health Sciences, Bethesda, Maryland, United States of America; Queensland Institute of Medical Research, Australia

## Abstract

Eukaryotes, protozoan, and helminth parasites make extensive use of protein kinases to control cellular functions, suggesting that protein kinases may represent novel targets for the development of anti-parasitic drugs. Because of their central role in intracellular signaling pathways, cyclic nucleotide–dependent kinases such as cAMP-dependent protein kinase (PKA) represent promising new targets for the treatment of parasitic infections and neoplastic disorders. However, the role of these kinases in schistosome biology has not been characterized and the genes encoding schistosome PKAs have not been identified. Here we provide biochemical evidence for the presence of a PKA signaling pathway in adult *Schistosoma mansoni* and show that PKA activity is required for parasite viability *in vitro*. We also provide the first full description of a gene that encodes a PKA catalytic subunit in *S. mansoni*, named SmPKA-C. Finally we demonstrate, through RNA interference, that SmPKA-C contributes to the PKA activity we detected biochemically and that inhibition of SmPKA-C expression in adult schistosomes results in parasite death. Together our data show that SmPKA-C is a critically important gene product and may represent an attractive therapeutic target for the treatment and control of schistosomiasis.

## Author Summary

Schistosomes are parasitic flatworms that inhabit the circulatory system and are the cause of a debilitating and insidious disease for millions of people worldwide. Like other complex organisms, schistosomes and other parasitic worms regulate their cell biology through extensive use of enzymes called protein kinases that phosphorylate other proteins to alter their function. One such protein kinase, cAMP-dependent protein kinase (PKA), has been proposed as a therapeutic target for the treatment of parasitic infections and cancer. Here we use biochemical techniques to show that schistosome worms possess a functional PKA pathway that is required for survival of the parasites. We also identify a parasite gene that encodes a functional PKA enzyme and show that silencing this gene results in both significant loss of PKA activity in schistosome worms and parasite death. These findings suggest that the gene we have identified is critically important to schistosomes and that its protein product may represent a target for the development of much-needed new drugs to treat schistosome infections.

## Introduction

Schistosomiasis, a disease caused by trematodes of the genus *Schistosoma*, afflicts approximately 200 million people in tropical and subtropical regions of the world and is responsible for approximately 280,000 deaths annually in Sub-Saharan Africa alone [1]. The schistosome life cycle is remarkably complex, involving multiple life cycle stages that are morphologically and physiologically adapted for survival within and transmission between the molluscan and vertebrate hosts these parasites require for life cycle completion. Within each host, evasion of host defenses is balanced with requirements for host resources and signals that are necessary for schistosome growth and development. While specific examples have not been characterized at the molecular level, there is considerable evidence for interactions between schistosomes and host factors, such as hormones and growth factors, which influence aspects of parasite biology such as development and reproduction [2]–[5]. This intimate relationship, where schistosomes exploit host factors to facilitate establishment of infection while simultaneously evading host defenses, is presumably a reflection of extensive host-parasite co-evolution that has occurred since the emergence of the genus *Schistosoma* some 12–19 million years ago [6].

Currently the anthelminthic praziquantel is the sole drug used for treatment of schistosomiasis, due to its ability to kill the adult worms of all medically important *Schistosoma* species [7]. However, there are reasons to suspect that reliance on this single drug for all treatment and control of schistosomiasis will not be sustainable in the long term. First, praziquantel-tolerant strains of *S. mansoni* can be derived in the laboratory by exposure to sub-curative doses of praziquantel [8],[9]. Second, evidence for decreased sensitivity to praziquantel has been found following mass drug treatment efforts [10]. Thus the potential for praziquantel resistance is real and the increasingly wide scale use of praziquantel, through programs such as the Schistosomiasis Control Initiative, highlight the necessity for the identification of new chemotherapeutic targets in schistosomes [11] .

Protein kinases represent a potentially new class of therapeutic targets for the treatment of parasitic diseases [12]. Through the phosphorylation of substrate proteins, protein kinases play a central role in the cellular signaling pathways of eukaryotic organisms and are involved in biological processes as diverse as gene expression, metabolism, apoptosis, and cellular proliferation [13]. The unregulated activity of protein kinases has been implicated in the pathogenesis of several human diseases, including cancer, autoimmune diseases and inflammation [14],[15]. Consequently, the development of protein kinase inhibitors as therapeutics for cancer and other diseases has been actively pursued [16]. As eukaryotes, protozoan and helminth parasites presumably also make extensive use of protein kinases to control cellular functions, suggesting that protein kinases may represent novel targets for the development of anti-parasitic drugs [17],[18]. Examples of promising protein kinase targets in parasites include the cyclic guanosine monophosphate- (cGMP-) dependent protein kinases (PKGs) of *Toxoplasma* [19], *Eimeria* [20] and *Plasmodium* [21], and a *Plasmodium* cyclic adenosine monophosphate- (cAMP-) dependent protein kinase (PKA) [22], as inhibition of these kinases resulted in significant anti-parasitic effects *in vivo* or *in vitro* [20],[23]. Regulated by cyclic nucleotide second messengers produced by purine nucleotide cyclases, cyclic nucleotide-dependent protein kinases represent particularly attractive drug targets as, in addition to targeting the kinase domain directly, their activity can also be manipulated by targeting the regulatory cyclic nucleotide binding (CNB) domains with cyclic nucleotide analogs [24]. Indeed, an experimental therapy for some cancers in which PKA is implicated utilizes this latter approach and is now in clinical trials [25]. A consistent difference between PKA and PKG across highly divergent taxa is that in PKA, the regulatory and catalytic activities are contained within separate gene products known as PKA-R and PKA-C respectively, whereas in PKG the CNB sites and catalytic domain are usually contained within the same polypeptide. Thus the inactive conformation of PKA is a heterotetramer of two PKA-R and two PKA-C subunits, while PKG exists as a homodimer. Segregation of the catalytic and regulatory functions of PKA into separate proteins provides an opportunity for diversification in the function of PKA, as different PKA-C and PKA-R isoforms can combine to produce holoenzymes with different functions [26]. Mammalian genomes contain as many as three *pka-c* genes and four *pka-r* genes, allowing for a variety of different holoenzymes to be formed [27].

While cyclic nucleotide-dependent kinases have been extensively characterized in a variety of eukaryotic organisms, including several parasites, there is relatively little data available on the role of these kinases in the biology of schistosomes. A study by Matsuyama et al. showed that treatment of schistosome miracidia with adenylyl cyclase and PKA inhibitors completely inhibited miracidial locomotion in a dose-dependent manner, suggesting a role for PKA in miracidial swimming [28]. In contrast, Kawamoto et al. found that treatment of miracidia with adenylyl cyclase agonists inhibited miracidium to mother sporocyst transformation, while drugs that decreased cAMP levels triggered transformation [29]. These studies suggest that cAMP and PKA play important roles in the larval stages of the schistosome life cycle. However, no studies have examined the role of PKA in adult schistosome biology and full-length nucleotide sequences encoding schistosome PKAs have not been identified. We hypothesized that PKA plays a vital role in adult worms and that targeting PKA may represent a novel approach to eliminating adult schistosomes from infected mammalian hosts. In this report, we provide a biochemical characterization and molecular identification of a *S. mansoni* PKA (SmPKA). Furthermore, we show that the schistosome PKA is an essential gene product for adult worms and as such represents an attractive therapeutic target for the treatment and control of schistosomiasis.

## Materials and Methods

### Ethics statement

All experiments involving mice were performed in accordance with protocols approved by the USUHS Institutional Animal Care and Use Committee.

### Parasite materials

*Biomphalaria glabrata* snails infected with NMRI/Puerto Rican strain of *S. mansoni* were supplied by Dr. Fred Lewis (Biomedical Research Institute, Rockville, MD). Cercariae were obtained by exposing infected snails to light for 2 h in 50 mL of filtered water. Schistosomula were prepared by mechanical transformation of cercariae according to published protocols [30]. Adult *S. mansoni* were obtained from 6 week-infected C57BL/6 mice that were infected with 150 cercariae using the tail immersion method [30].

### Western blotting

Freshly isolated adult worms were homogenized in cell extraction buffer (100 mM NaCl, 25 mM Tris pH 7.5) containing a protease inhibitor cocktail (Sigma; 4-(2-aminoethyl) benzenesulfonyl fluoride hydrochloride (AEBSF; 1 mM), aprotinin (0.8 µM), leupeptin (20 µM), bestatin (40 µM), pepstatin A (15 µM), and E-64 (14 µM)). The resulting homogenate was incubated on ice for 30 min and centrifuged at 13,000 rpm for 20 min at 4°C to remove insoluble material. The protein concentration of the resulting supernatant (Sm lysate) was determined using the Quick Start Bradford Protein Assay. Western blots were performed using the WesternBreeze Chemiluminescent Western Blot Immunodetection Kit (Invitrogen). Briefly, 7 µg of total protein from adult worms, HT1080, and 293FT cells were used per sample. Reduced samples were separated by SDS-PAGE on 12% Bis-Tris gels and transferred onto polyvinyl difluoride (PVDF) membranes. After an initial blocking step in 5% non-fat dried milk, 20 mM Tris pH 7.5, membranes were incubated for 12 h with polyclonal anti-PKA C-α antibody (Cell Signaling Technology) diluted 1∶1000 and then with alkaline phosphatase-conjugated goat anti-rabbit secondary antibody (Invitrogen) for 2 h. Bound antibody was detected according to manufacturer's instructions using Kodak BioMax Light Film.

### Detection of PKA enzymatic activity

PKA activity was measured using a fluorescent peptide substrate-based assay (Omnia Lysate Assay for PKA kit, Biosource). Freshly isolated adult worms were used to prepare Sm lysate, in the presence of protease and phosphatase inhibitor cocktails (Phosphatase Inhibitor Cocktail 1(Sigma), containing cantharidin, bromotetramisole and microcystin LR, diluted 1∶100) as described above. The protein concentration was determined using the Quick Start Bradford Protein Assay and adjusted to a final concentration of 0.2 µg/μL with additional extraction buffer. Kinase reactions containing 1 µg total protein (equivalent to 5 µL Sm lysate), 10 mM ATP, 15 µM PKA peptide substrate, 10 mM DTT and a non-PKA inhibitor cocktail (64 µM PKC inhibitor peptide, 10 µM GF109203X, 20 µM calmidazolium) in kinase reaction buffer were assembled in opaque 96-well assay plates, according to the manufacturer's recommendations. Accumulation of phosphorylated substrate was monitored during a 1 h incubation at 30°C by recording fluorescent emissions at a wavelength of 485 nm upon excitation at 360 nm, using a Spectramax M2 microplate fluorometer (Molecular Devices). Fluorescence measurements were recorded every 30 s in relative fluorescence units (RFUs). Kinase reactions containing 2 ng recombinant human PKA-Cα catalytic subunit (Invitrogen) were used as positive controls. PKA activity was plotted using GraphPad Prism software version 4 (Graphpad Software, Inc.). Symbols at each time point represent the means of three biological replicates and experiments were performed at least twice.

### PKA inhibitor assays

H-89 and protein kinase A inhibitor fragment 14–22 (PKI 14–22 amide) were purchased from Invitrogen. H-89 was dissolved in dimethyl sulfoxide (DMSO) and PKI 14–22 amide was dissolved in water. Kinase reactions were performed as described above in the presence of H-89 and PKI 14–22 amide or appropriate vehicle control at the following concentrations: 500, 100, and 10 µM. The maximum concentration of DMSO in any reaction was less than 5% and no differences in kinase activity were observed between controls treated with water or DMSO. PKA activity in presence and absence of inhibitor was determined as described in the previous section.

### Adenylyl cyclase agonist and inhibitor assays

Forskolin and SQ22536 were purchased from Sigma and stock solutions prepared in DMSO. To examine the effects of forskolin and SQ22536 on PKA activity, triplicate groups of freshly isolated adult worm pairs (10 pairs per group) were incubated for 2 h at 37°C in 24 well tissue culture plates containing Dulbecco's modified Eagle's medium (DMEM) in the presence of 100 or 50 µM of forskolin, SQ22536 or DMSO alone. Then Sm lysate was prepared from the treated worms and PKA activity was measured as described above.

### *In vitro* treatment of adult worms with PKA inhibitors

Effects of SmPKA inhibition in adult worms were assessed using H-89 and PKI 14–22 at the following concentrations: 500, 250, 100, 50, 25, 10, and 1 µM. Individual adult worm pairs (6 pairs per concentration) were placed in the wells of 24 well tissue culture plates containing 1 mL total of DMEM (with 10% fetal bovine serum and 5% penicillin/streptomycin) and appropriate concentration of inhibitor. Equal amounts of appropriate vehicle alone were added to the wells containing control worms. Medium containing inhibitor or vehicle was replaced daily. Worms were incubated at 37°C in 5% CO_2_ and observed every 24 h for a period of 7 d. Worms were considered to be dead when all evidence of motility, including gut peristalsis, had ceased. Kaplan-Meier survival curves were generated using GraphPad Prism software. Photomicrographs were obtained using a Zeiss CL1500ECO dissecting microscope.

### SmPKA-C cDNA cloning and sequence analysis

Total RNA was extracted from adult worms using the RNAzol B Method (IsoTex Diagnostics, Inc.). 1 µg RNA was used to synthesize cDNA using the iScript Select cDNA Synthesis Kit and an oligo (dT)_20_ primer (Bio-Rad). The full-length cDNA sequence of SmPKA-C was obtained using the RNA ligase-mediated rapid amplification of 5′ and 3′ cDNA ends (RACE) kit (Invitrogen) and internal gene specific primers designed from the *S. mansoni* EST Sm11052. RACE products of interest were purified using the QIAquick Gel Extraction Kit (Qiagen), cloned into the pCR2.1-TOPO vector (Invitrogen) and sequenced using the Big-Dye Terminator cycle sequencing kit (Applied Biosystems). The SmPKA-C cDNA sequence was compared to genomic sequences available at the National Center for Biotechnology Information (http://blast.ncbi.nlm.nih.gov/Blast.cgi) and the *S. mansoni* Genome Project website (http://www.genedb.org/genedb/smansoni/blast.jsp) blast servers. Vector NTI software (Invitrogen) was used to align the nucleotide and amino acid sequences of SmPKA-C with PKA-C subunit sequences from other eukaryotic organisms. Phylip (Phylogeny Inference Package) software [31] was used to construct the phylogenetic tree using the protein parsimony method (http://mobyle.pasteur.fr/cgi-bin/portal.py?form=protpars).

### Detection of SmPKA-C gene expression in *S. mansoni* life cycle stages

Total RNA was extracted and cDNA was synthesized from male and female adult worms, schistosomula, and cercariae as described above. Egg, miracidium, and sporocyst cDNA were kindly provided by Dr. Conor Caffrey (Sandler Center for Basic Research in Parasitic Diseases, San Francisco, CA). To detect SmPKA-C expression, the following primers were used to amplify a 900 bp fragment of the SmPKA-C cDNA (nucleotide positions 50–952): forward 5′–GGTAATGCACAAGCTGCTAAA–3 and reverse 5′-CCAATCGGTTGTTGCAAACC-3′. The *S. mansoni* alpha tubulin cDNA (GenBank Accession No. S79195) was used as a positive control and a 100 bp fragment (nucleotide positions 1711–1824) was amplified by PCR using the following primers: forward 5′-GGTTGACAACGAGGCCATTTATG-3′ and reverse 5′-TGTGTAGGTTGGACGCTCTATATCT-3′. Amplicons were visualized by agarose gel electrophoresis.

### SmPKA-C gene silencing using RNAi

A 900 bp SmPKA-C cDNA fragment was generated by PCR using the primers and cycling parameters described above and the resulting amplicon was cloned into pCRII-TOPO vector (Invitrogen). Plasmid DNA was linearized using *Sac*I or *Xho*I and used as template to transcribe ssRNA using T7 and Sp6 RNA polymerases and the MEGAscript RNA transcription kit (Ambion) [32]. SmPKA-C dsRNA was generated and purified using the MEGAscript RNAi kit (Ambion) according to the manufacturer's instructions. A non-schistosome control dsRNA was generated from the pCR-II TOPO vector as described above. Integrity of the final dsRNA products was assessed by agarose gel electrophoresis. 10 or 30 µg of SmPKA-C or control dsRNA, diluted in 100 µl of electroporation buffer (Ambion), were delivered to groups of 12 mixed-sex adult worms via electroporation as described previously [33]. Adult worms, placed in 4 mm cuvettes, were pulsed at room temperature with a single 20 ms square wave pulse at 125 V using the GenePulser Xcell Electroporation system (Bio-Rad). Adult worms were immediately transferred to pre-warmed DMEM (10% FBS and 5% penicillin/streptomycin) and maintained at 37°C. To assess the efficacy of SmPKA-C RNA knockdown, RNA was extracted after 3 d and transcript levels assessed by PCR as described above, except that primers which hybridize outside the targeted region were used (forward 5′-CGCGTAATATCACTTGAGAGTCAAAATAG-3′ and reverse 5′-AAATTCACTAAATTCTTTTGCACATTTCTCTGTTGTAGCAATACG-3′, to amplify a fragment corresponding to nucleotide positions 18–1096), and the accumulation of PCR product was monitored in real time by detection of SYBR Green fluorescence, using a M.J. Research Chromo4 PCR cycler (Bio-Rad). Relative SmPKA-C RNA levels were calculated using the 2^−ΔΔCt^ method [34] and *S. mansoni* alpha tubulin RNA as the control transcript. Data are representative of two independent experiments.

### Statistical analyses

The statistical significance of differences between treated and control groups in activity assays was calculated using one-way ANOVA of repeated measures. The statistical significance of differences between Kaplan-Meier survival curves was calculated using the logrank test. *P* values≤0.05 were considered statistically significant. Student's T test with Welch's correction was used to test the significance of differences in expression levels detected by real-time PCR. GraphPad Prism software was used for all statistical analyses.

## Results

### Detection of PKA protein expression and PKA enzymatic activity in adult *S. mansoni*

**Figure 1:**
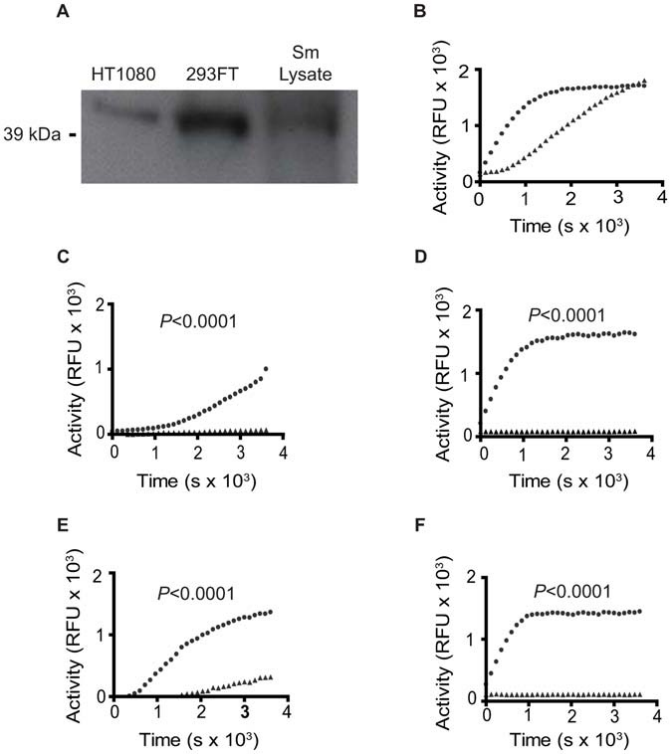
Detection of a putative schistosome PKA in parasite lysates. A, Western blotting of lysates from adult *S. mansoni* (Sm lysate) and two human cell lines (293FT, HT1080) using a polyclonal antibody specific for the human PKA-Cα subunit revealed the presence of a putative PKA-C subunit of the expected molecular weight of 40 kDa in the schistosome lysate. B, Putative PKA activity is detectable in lysate of adult *S. mansoni* worms using a fluorescence-based kinase assay. ▴, Sm lysate, • recombinant human PKA-Cα. C–F, Kinase activity was measured in kinase reactions containing Sm lysate (C and E) and recombinant human PKA-Cα (D and F) and the PKA inhibitors H-89 (C and D) and PKI 14–22 (E and F). ▴, inhibitor-treated; •, no inhibitor. B–F, each time point represents the mean of three biological replicates.

To detect putative PKA-C subunit homologues in adult *S. mansoni* protein lysate (Sm lysate), western blot analysis using a polyclonal antibody generated against a conserved epitope of the C-terminus of human PKA-Cα subunit was conducted (GenBank Accession No. P17612). A band of 40 kDa was detected in Sm lysate, which was similar in molecular weight to the human PKA-Cα detected in the two human cell lines (Fig. 1A).

We next sought to determine if protein extracts from adult *S. mansoni* had measurable PKA activity. Using a fluorescent peptide substrate-based assay, a putative PKA activity was detected in adult Sm lysate, as determined by the accumulation of a fluorescent, phosphorylated peptide reaction product (Fig. 1B). While reaction product accumulated more slowly in the Sm lysate reaction than in a positive control reaction containing recombinant human PKA-Cα (hereafter referred to as control PKA), similar total amounts of product accumulated in both reactions by the end of the assay.

While the peptide substrate used in the kinase activity contains a specific PKA target sequence, other protein kinases such as protein kinase C (PKC) and calmodulin-dependent protein kinases (CDPKs) share similar substrate specificity to PKA [35]. For this reason, a kinase inhibitor cocktail that inhibits PKC and CDPK was included in all assays to eliminate non-PKA mediated phosphorylation. To further confirm that the kinase activity detected in Fig. 1B was attributable to a PKA enzyme, adult Sm lysate was treated with inhibitors that target PKA-C subunits, H-89 and PKI 14–22 amide, and the resulting activity was compared to PKA activity in untreated control lysate. The PKA activity of adult Sm lysate and control PKA was completely inhibited by 10 µM H-89 (*P*<0.0001) (Figs. 1C and 1D), an ATP-competitive inhibitor that is a potent inhibitor of PKA both *in vitro* and *in vivo* [36]. Identical results were obtained with 100 and 500 µM H-89 (data not shown). Similar to treatment with H-89, 10 µM PKI 14–22 amide also significantly inhibited the PKA activity of Sm lysate and the control PKA (*P*<0.0001) (Figs. 1E and 1F). PKI 14–22 amide is a highly specific inhibitor of PKA as it contains a pseudosubstrate site which facilitates high affinity binding to the substrate binding site of PKA-C subunits, preventing docking of the substrate [37],[38].

### Modulation of parasite PKA activity through manipulation of cAMP

**Figure 2:**
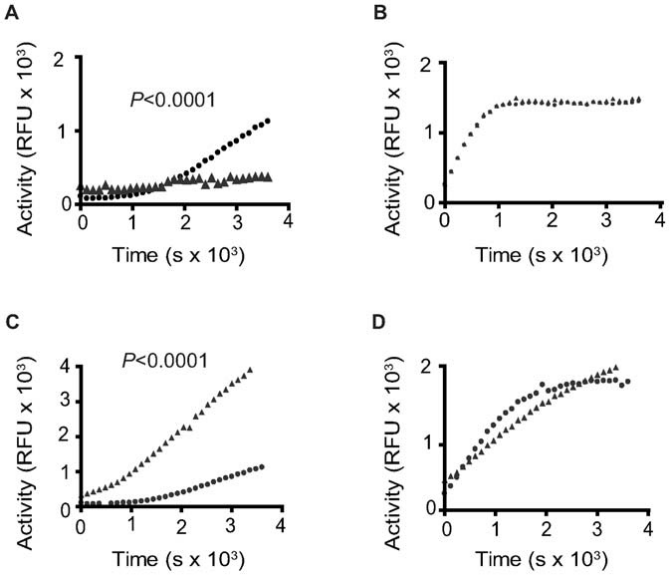
Schistosome PKA activity is sensitive to adenylyl cyclase modulation. Kinase activity was measured in kinase reactions containing Sm lysate from adult worm pairs (A and C) that were previously treated with SQ22536 (A) or forskolin (C). Treatment groups contained 10 worm pairs (20 worms total) each and were performed in triplicate. Kinase reactions containing recombinant human PKA-Cα (B and D) were treated directly with SQ22536 (B) or forskolin (D). ▴, inhibitor-treated; •, no inhibitor. Data are representative of two independent experiments.

Since cAMP binding to PKA-R subunits is required for PKA-C release and activation, we hypothesized that if the kinase activity we detected in Sm lysate was attributable to a PKA, its activity would be sensitive to alterations in the availability of cAMP for R subunit binding. To test this hypothesis, we tested whether the schistosome PKA activity was sensitive to manipulation of endogenous adenylyl cyclase activity [39]. The adenylyl cyclase inhibitor, SQ22536 [40], and adenylyl cyclase agonist, forskolin [41], were each used to either inhibit or activate endogenous adenylyl cyclase activity, respectively. In order to maintain the integrity of intracellular signaling pathways in these experiments, intact adult worms were treated with inhibitor or agonist rather than Sm lysate, as cellular structure is lost during preparation of the parasite lysate. 100 µM SQ22536 significantly decreased PKA activity in treated adult *S. mansoni* worms when compared to the untreated controls (*P*<0.0001) (Fig. 2A), presumably by inhibiting cAMP production and preventing the disassociation of PKA-C subunits from the holoenzyme. In contrast to SQ22536, 100 µM forskolin significantly increased PKA activity in treated adult worms as compared to the untreated controls (*P*<0.0001) (Fig. 2C). Similar activation and inhibition were seen with 50 µM of forskolin and SQ22536, respectively (data not shown). As expected, the activity of the recombinant control PKA preparation was not affected by SQ22536 or forskolin, as this preparation does not contain adenylyl cyclase or PKA-R subunits (Figs. 2B and 2D).

Taken together, these data further support the conclusion that the kinase activity we detected in adult *S. mansoni* extracts is attributable to a PKA enzyme, as this activity can be inhibited and activated using inhibitors and agonists of adenylyl cyclase. Furthermore, our data suggest that *S. mansoni* possesses a functional cAMP signaling pathway, containing adenylyl cyclase and both regulatory and catalytic PKA subunits.

### PKA activity is required for the viability of adult *S. mansoni* worms *in vitro*

**Figure 3:**
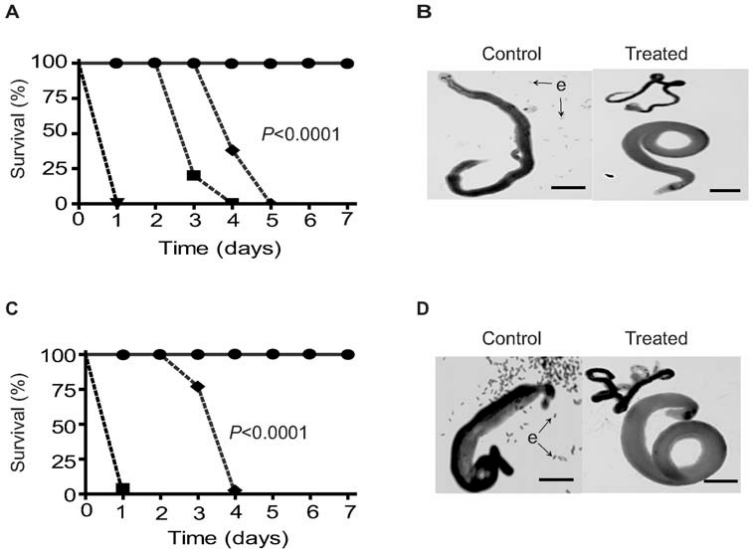
PKA activity is essential for adult *S. mansoni* viability *in vitro*. Adult *S. mansoni* worm pairs were maintained in medium containing varying concentrations of H-89 (A and B) or PKI 14–22 amide (C and D). Survival in the presence of each inhibitor was plotted against time (A and C). For H-89, concentrations of inhibitor are as follows: 0 and 1 µM (•); 10 µM (♦); 25 µM (▪); 50, 100, 250, and 500 µM (▾). For PKI 14–22 amide concentrations of inhibitor are as follows: 0, 1, 10, 25, and 50 µM (•); 100 µM (♦); 250 and 500 µM (▪). Treatment groups containing 6 worm pairs each (12 worms total) were used for each concentration. B and D, micrographs of representative worm pairs incubated in the presence of 100 µM H-89 (B) or100 µM PKI 14–22 amide (D). Scale bars = 1 mm. e, parasite eggs. Data are representative of three independent experiments.

To test whether the schistosome PKA activity plays a significant role in parasite biology, we next analyzed the effect of the inhibitors H-89 and PKI 14–22 amide on adult worms *in vitro*. Treating worms with H-89 at concentrations of 50–500 µM resulted in 100% mortality within 24 h (Fig. 3A). Incubation of worms with H-89 at 25 µM resulted in 75% mortality by Day 3 and 100% by Day 4 (Fig. 3A). Treatment with H-89 at 10 µM resulted in 100% mortality by Day 5 (Fig. 3A). Prior to parasite death, exposure to H-89 caused a cessation in egg production and resulted in dissociation of males and females (Fig. 3B), effects that were evident at H-89 concentrations as low as 1 µM concentration (data not shown), despite the lack of a killing effect (Fig. 3A). Similarly, incubation with 500 and 250 µM of PKI 14–22 amide resulted in 100% worm mortality within 24 h of exposure (Fig. 3C), while at 100 µM some worms survived until Day 3 (Fig. 3C). Incubating worms with 1–50 µM PKI 14–22 amide resulted in 100% survival (Fig. 3C). As with H-89, PKI 14–22 amide caused cessation of egg production and unpairing prior to parasite death (Fig. 3D). These data show that loss of PKA activity by inhibition of PKA-C subunits is lethal for adult *S. mansoni in vitro* and suggest that the schistosome PKA is essential for maintaining parasite viability.

### Identification of a SmPKA-C cDNA and its expression in *S. mansoni* life cycle stages

**Figure 4:**
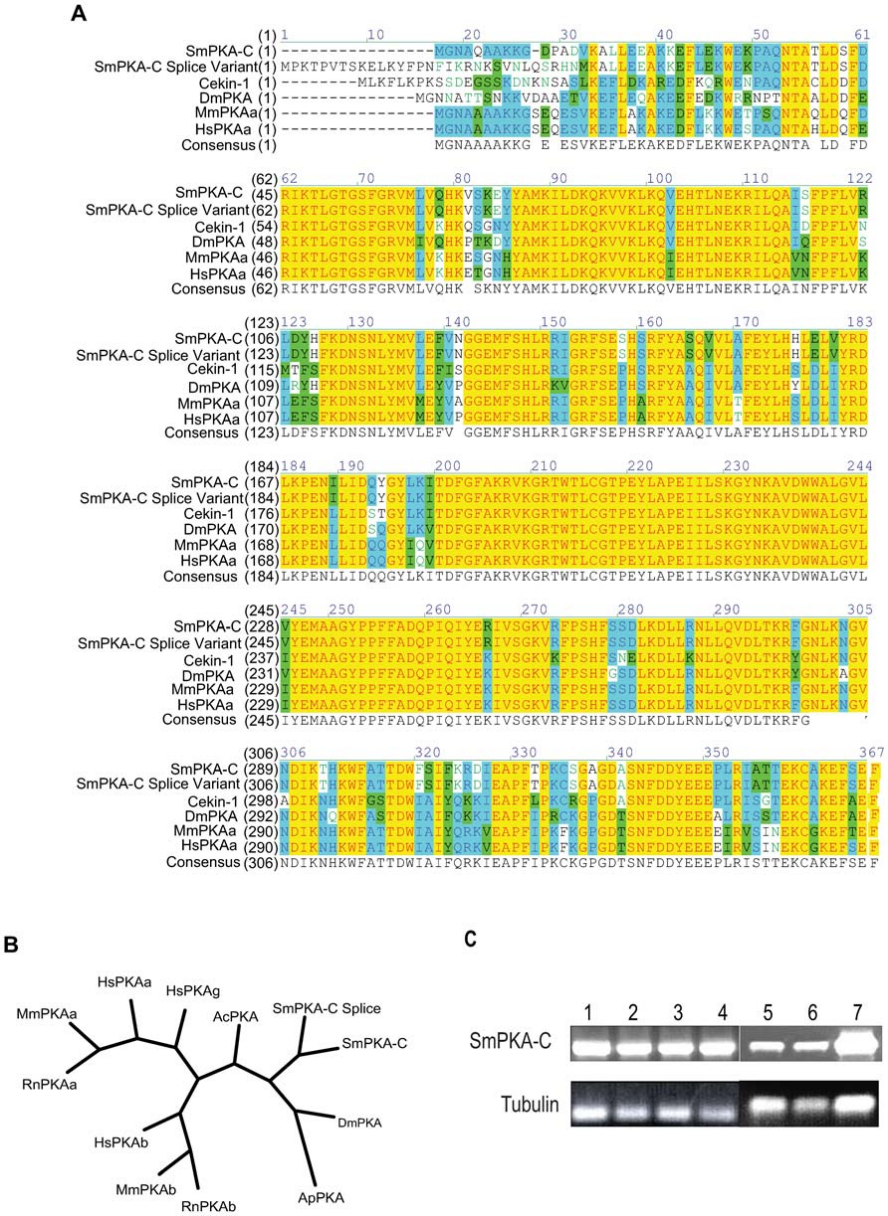
Identification of SmPKA-C as a PKA-C subunit from *S. mansoni*. A, Amino acid alignment of both SmPKA-C and putative splice variant peptide sequences with PKA-C sequences from *Caenorhabditis elegans* (Cekin-1; NP_493605), *Drosophila melanogaster* (DmPKA; NP_476977), *Mus musculus* (MmPKAa; P05132) and *Homo sapiens* (HsPKAa; P17612). B, Phylogenetic analysis comparing both SmPKA-C amino acid sequences to the following PKA-C sequences from other organisms: *Homo sapiens* PKA-Cα (HsPKAa; P17612), *H. sapiens* PKA-Cβ (HsPKAb; P22694), *H. sapiens* PKA-Cγ (HsPKAg; P22612), *Mus musculus* PKA-Cα (MmPKAa; P05132), *M. musculus* PKA-Cβ (MmPKAb; P68181), *Rattus norvegicus* PKA-Cα (RnPKAa; P27791), *R. norvegicus* PKA-Cβ (RnPKAb; P68182), *D. melanogaster* (DmPKA; NP_476977), *Aplysia californica* (AcPKA; CAA45014), and *Acyrthosiphon pisum* (ApPKA; XP_001946114). C, SmPKA-C transcript is expressed in cDNA of all the life cycle stages of *S. mansoni*. 1, egg, 2, miracidium, 3, sporocyst, 4, cercaria, 5, schistosomulum, 6, adult female, 7, adult male.

To identify cDNA sequences that might encode for PKA-C subunits in *S. mansoni*, BLASTX searches of the *S. mansoni* genome database (http://www.genedb.org/genedb/smansoni/blast.jsp) were performed using PKA-C protein sequences from other organisms. A 622 bp EST (Sm11052) with significant similarity to other PKA-C subunits was identified. Sm11052 contained an incomplete open reading frame (ORF) that encoded for the N-terminal portion of a protein kinase domain, as determined by the presence of a complete ATP-binding site (corresponding to the consensus motif Gly–x–Gly–x–x - Gly–x–Val) and a serine/threonine kinase active site containing the motif Arg - Asp–Asp–Leu–Lys–x–x–Asn [13]. The complete sequence of the cDNA was obtained by 5′ and 3′ RACE using gene-specific primers that annealed within the Sm11052 sequence and the entire cDNA was then amplified from adult *S. mansoni* cDNA. The full-length cDNA is 1899 bp long and contains a complete ORF of 1053 bp, encoding for a protein of 350 amino acids in length and with a predicted molecular mass of 40.4 kDa (GenBank Accession No. GQ168377). The ORF encoded for a putative protein kinase, with intact N and C-termini and a C-terminal kinase domain that contained all 12 conserved subdomains characteristic of protein kinase domains [13]. BLAST comparison of the amino acid sequence with the non-redundant protein sequence database at NCBI showed that the putative *S. mansoni* PKA-C (SmPKA-C) protein shared 70% similarity with PKA-C subunits from other eukaryotic organisms (*Caenorhabditis elegans, Drosophila melanogaster, Mus musculus*, and *Homo sapiens*) (Fig. 4A) and was most similar to the PKA-C subunit from *Aplysia californica* (Fig. 4B). The estimated molecular mass of SmPKA-C protein was also similar to that of other PKA-C proteins and approximately matched the apparent mass of the band detected in *S. mansoni* extracts by western blot using anti-human PKA-C antibodies in Fig. 1A. Qualitative analysis of SmPKA-C expression in various life cycle stages by reverse transcriptase PCR revealed that SmPKA-C transcript was detectable in all *S. mansoni* life cycle stages tested (egg, miracidium, sporocyst, cercaria, schistosomulum, adult male and female; Fig. 4C).

The ORF of SmPKA-C was then compared using BLAST analysis to the *S. mansoni* genome database to identify other putative PKA-C subunit sequences encoded by the *S. mansoni* genome. One sequence, Smp_152330, was identified that was 95% identical to the SmPKA-C nucleotide sequence. PCR analysis showed that the 3′end of the predicted database sequence was incorrect and, using 3′ SmPKA-C gene-specific primers, we were able to amplify the correct cDNA sequence of Smp_152330 from adult *S. mansoni* cDNA. Translation of the Smp_152330 nucleotide sequence showed it contained 18 more amino acids at the N-terminus than the SmPKA-C protein, but the remainder of both the nucleotide and amino acid sequences were identical (Fig. 4A and data not shown), suggesting that the corrected Smp_152330 sequence represents an alternatively spliced form of SmPKA-C.

### Silencing of SmPKA-C expression by RNAi

**Figure 5:**
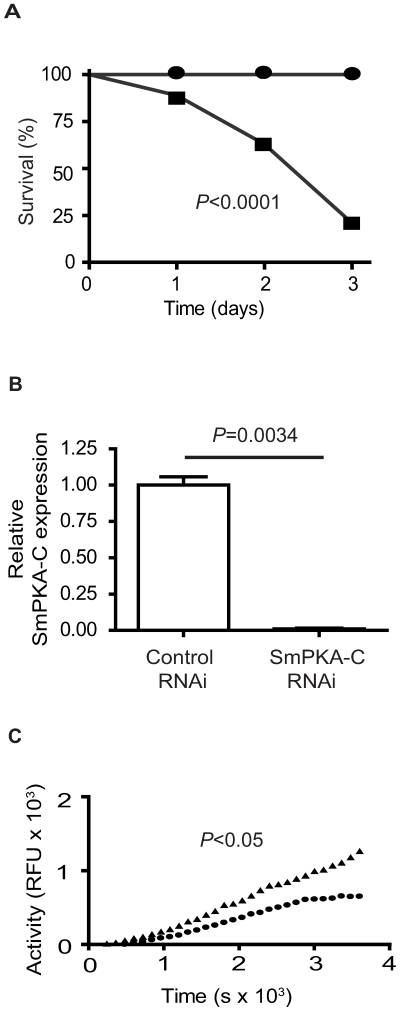
SmPKA-C encodes for an essential kinase activity. A, Electroporation of adult worms with 30 µg of SmPKA-C dsRNA (▪) resulted in 75% mortality while worms treated with control dsRNA (•) exhibited 100% survival. B, Three days after electroporation, SmPKA-C transcript levels were decreased in worms electroporated with 10 µg of SmPKA-C dsRNA, while transcript levels were unaffected in control dsRNA-treated worms, as determined by real-time PCR. C, 7 days after electroporation, SmPKA-C dsRNA-treated (•) and control dsRNA-treated worms (▴) were incubated for 2 h in 100 µM forskolin. PKA activity was significantly decreased after electroporation with 10 µg SmPKA-C dsRNA compared to control dsRNA-treated worms.

To determine the effect of silencing SmPKA-C expression in *S. mansoni*, adult worms were treated via electroporation with 30 µg of SmPKA-C dsRNA or control dsRNA. We observed 75% mortality of adult worms treated with 30 µg of SmPKA-C dsRNA by Day 3, while all control dsRNA-treated parasites survived (Fig. 5A). To reduce parasite mortality and provide more surviving worms for subsequent analysis, the amounts of dsRNA were reduced in subsequent experiments. Treatment with 10 µg of SmPKA-C dsRNA resulted in 92% survival of worms, while treatment with 10 µg of control dsRNA again resulted in 100% survival to Day 3 (data not shown). Real-time PCR analysis of SmPKA-C transcript levels in surviving worms on day 3 post-electroporation revealed a significant reduction of SmPKA-C mRNA in SmPKA-C dsRNA-treated worms to approximately 1% of the levels detected in control dsRNA-treated worms (*P* = 0.0034) (Fig. 5B).

To determine whether SmPKA-C contributes to the PKA activity detected in Sm lysate, PKA activity was measured in lysates prepared from worms that were treated with 10 µg SmPKA dsRNA or control dsRNA 7 days previously. Worms were treated with 100 µM forskolin for 2 hours prior to lysate preparation in order to reactivate PKA activity. SmPKA activity was significantly reduced in SmPKA-C dsRNA treated worms as compared to the control dsRNA treated worms (*P*<0.05) (Fig. 5C).

## Discussion

In this study, we provide the first direct evidence for the expression of an active PKA in adult *S. mansoni* worms. First, antibodies to a highly conserved motif from the PKA-C kinase domain of other organisms reacted with a protein of the expected size for a PKA-C subunit in protein extracts from adult worms. Second, a protein kinase assay utilizing a peptide substrate that is preferentially phosphorylated by PKAs demonstrated significant PKA kinase activity in adult worm lysates. Third, the activity of the putative PKA was sensitive to known inhibitors of PKA-C kinase activity, providing further evidence that a PKA-C protein was responsible for the activity we detected in parasite extracts. Finally, exposure of intact parasites to an adenylyl cyclase inhibitor and agonist either decreased or increased, respectively, the PKA activity, demonstrating that the putative parasite PKA exhibits the expected sensitivity to modulation of cAMP levels. Taken together, these data support the conclusion that an active PKA is expressed in adult *S. mansoni*, and that adult schistosomes also express regulatory proteins that control PKA activation by cAMP, such as adenylyl cyclase and PKA regulatory subunits.

We are aware of one other report which demonstrated that targeting protein kinases in adult schistosomes can be detrimental to the parasites. In this report, the authors demonstrated that, while exposure of adult *S. mansoni* worms to the broad spectrum tyrosine kinase inhibitor herbimycin A *in vitro* was not lethal to schistosome worms, parasite egg production was significantly inhibited in a dose-dependent manner [42]. Here we demonstrate that exposure of intact adult worms to inhibitors of PKA kinase activity is lethal to the parasites, suggesting that, in contrast to herbimycin-sensitive tyrosine kinases, the schistosome PKA is critically important for viability. H-89 in particular killed schistosomes rapidly at low concentrations *in vitro*. However, while H-89 is considered a specific inhibitor of PKA and its IC_50_ for PKA is in the low nanomolar range, it has also been shown to inhibit other protein kinases. For example, previous studies showed that H-89 also inhibited ribosomal protein S6 kinase 1 (S6K1) and mitogen-and stress-activated protein kinase (MSK1) at lower concentrations than PKA [36],[43]. Since these two kinases play a major role in eukaryotic cell biology, the lethality observed on treating adult worms with H-89 may not be due to PKA inhibition alone, but rather the result of inhibition of several other kinases in addition to PKA. However PKI 14–22 amide, a highly specific inhibitor of PKA-C subunits that does not affect other protein kinases [37], also caused parasite death, albeit at higher concentrations than H-89, supporting the conclusion that PKA activity is important for maintaining parasite viability. A possible explanation for the difference in parasite killing we observed with these two inhibitors is that PKI 14–22 amide is a peptide, which would not be expected to penetrate the treated parasites as well as H-89. Alternatively, inhibition of additional kinases by H-89 may enhance its toxicity for schistosome worms.

Partial coding sequences for putative PKA-C genes have been generated by the *S. mansoni* genome project, but no full-length sequences for a schistosome PKA-C have been identified. Here we report the isolation of a full-length cDNA encoding for a *S. mansoni* PKA-C subunit we named SmPKA-C. Expression of this transcript was detected in all life cycle stages we examined, including adults, suggesting this cDNA may encode the PKA activity we detected in adult worm extracts. Subsequent targeting of the SmPKA-C transcript in adult schistosomes using RNAi was lethal for the parasites, demonstrating that this gene is essential for parasite viability, at least *in vitro*, and providing further support for the conclusion that the parasite death we observed with the inhibitors H-89 and PKI 14–22 amide were the result of PKA inhibition. Consistent with this conclusion, RNAi inhibition of SmPKA expression resulted in significant loss of PKA activity in parasite lysates, confirming that at least a portion of the PKA activity in adult worms is encoded by this transcript. Interestingly, not all PKA activity was ablated by RNAi of SmPKA, despite significant knock-down of transcript levels to approximately 1% of the levels observed in control worms, raising the possibility that other PKA isoforms are expressed by adult worms. Alternative splicing in the N-termini of PKA-C subunits has been observed in mammalian species and in invertebrates, such as *C. elegans* [44],[45], and our RACE experiments suggest that alternative splicing results in the expression of at least two SmPKA-C isoforms in adult schistosomes. However, the remaining activity cannot be attributed to either of these splice variants, as the two transcripts share the sequence targeted by the dsRNA fragment we used for RNAi. These observations suggest that *S. mansoni* expresses other PKA isoforms, perhaps encoded by additional PKA genes or generated by additional, less conservative alternative splicing, and argue that a search for additional PKA-encoding sequences in *S. mansoni* mRNA and genomic DNA is warranted. Alternatively, the remaining PKA activity detected in RNAi-treated worms may be due to residual SmPKA-C expression, as ablation of SmPKA-C transcript was not absolute (Fig. 5B). Interestingly we were unable,to amplify full-length cDNAs from adult worms that correspond to other putative PKA genes that have been predicted from sequences of *S. mansoni* genomic DNA (Smp_080770, Smp_047150.2/1), suggesting that these genes are not expressed in adult schistosomes or that the predicted coding sequences are incorrect. Other putative *S. mansoni* PKA sequences (Smp_147450 and Smp_194610) appear to lack a functional kinase domain or are missing amino acid residues that are critical for PKA function. Thus the extent to which adult schistosomes express multiple PKA isoforms remains unclear. However, as RNAi knockdown of SmPKA-C resulted in parasite death, we conclude that SmPKA-C is a critically important protein and propose that SmPKA may be an attractive target for the development new schistosomicidal therapeutics.

Tight regulation of PKA activity by PKA-R subunits provides additional opportunities for pharmacological manipulation of PKA, beyond targeting the PKA-C subunit directly with kinase inhibitors. To date, there are no published reports that identify or characterize PKA-R subunits in schistosomes, but the cDNA sequence of a putative PKA-R subunit from *S. mansoni* was recently released by the *S. mansoni* genome project (GenBank accession no. CAY17207) and others may await identification. In other organisms, variation in CBD sequences amongst PKA-R isoforms results in differential affinity for cAMP and for cAMP analogs that either induce or inhibit holoenzyme dissociation and activation of the PKA-C subunits [46],[47]. Thus there is considerable potential for pharmacological manipulation of PKA activity using cAMP analogs. One such analog, 8-Cl-cAMP, has been shown to be a potent growth inhibitor in numerous human cancer cell lines and has completed phase I clinical trials for the treatment of some cancers [25]. Identification and analysis of schistosome PKA-R subunits may identify opportunities for the pharmacological targeting of parasite PKA in a similar manner to that now in development for cancer treatment. These observations highlight the obvious parallels between the treatment of cancer and parasitic infections, which both involve the targeting of eukaryotic cells, and suggest that novel approaches to cancer chemotherapy may provide new leads for the development of much needed anti-parasitic drugs.
